# Safety and effectiveness of cannabinoids to Danish patients with treatment refractory chronic pain—A retrospective observational real‐world study

**DOI:** 10.1002/ejp.2054

**Published:** 2022-12-15

**Authors:** Tina Horsted, Karoline Lichon Hesthaven, Peter Derek Christian Leutscher

**Affiliations:** ^1^ The Pain Clinic in Copenhagen, Horsted Institute Copenhagen Denmark; ^2^ Centre for Clinic Research, North Denmark Regional Hospital Denmark; ^3^ Department of Clinical Medicine Aalborg University Aalborg Denmark

## Abstract

**Background:**

Cannabinoids are considered a therapeutic option to patients suffering from treatment refractory chronic pain (TRCP) insufficiently relieved by conventional analgesics or experiencing intolerable adverse events (AEs) from those. This study aimed to explore safety and effectiveness of oral cannabinoids among patients with TRCP.

**Methods:**

A retrospective study was conducted among Danish patients with TRCP being prescribed oral cannabinoids. Data on AEs and changes in pain intensity by numeric rating scale (NRS) before and after initiation of oral cannabinoid therapy were analysed.

**Results:**

Among 826 eligible patients ≥18 years old, 529 (64%) were included for data analysis at first follow‐up (F/U1) (median 56 days from baseline) and 214 (26%) for second follow‐up (F/U2) (median 126 days from F/U1). Mean age was 60 ± 15.9 years and 70% were females. AEs were in general reported mild to moderate by 42% of patients at F/U1 and 34% at F/U2. AEs were mainly related to gastrointestinal (F/U1: 17% and F/U2: 13%) and nervous system disorders (F/U1: 14% and F/U2: 11%). Reduction in NRS was significantly different at both follow‐up consultations compared with baseline (<0.0001). Clinically relevant pain reduction (NRS ≥30%) was reported by 17% at F/U1 and 10% of patients at F/U2 in *intention‐to‐treat* analysis whereas the figures were 32% and 45% respectively, in *per‐protocol* analysis.

**Conclusion:**

Oral cannabinoid therapy seems to be safe and mildly effective in patients with TRCP. Randomized controlled trials with focus on comparable pain characteristics in diagnostical homogenous patient subgroups are needed for further improvement of evidence level for relief of chronic pain using oral cannabinoids.

**Significance:**

The findings in this retrospective study conducted in a real‐world clinical setting suggest a favourable safety profile of cannabinoids. Moreover, one‐sixth (intention‐to‐treat) and one‐third (per‐protocol) of patients with chronic pain refractory to conventional analgesics, or experiencing intolerable adverse effects, benefited significantly from therapy with oral cannabinoid regimens. Combination of THC and CBD seems overall more effective than cannabinoid monotherapy. Conduction of randomized controlled trials investigating safety and efficacy of cannabinoid therapy to diagnosis specific patient subgroups with comparable clinical and pathophysiological chronic pain characteristics is warranted, hence contributing further to the process of clinical evidence clarification currently in progress.

## 1 INTRODUCTION

Different medical conditions may cause manifestation of chronic pain, negatively affecting patients physically, mentally and socially (Finnerup et al., 2015; Harker et al., 2012; Gerhart et al., 2017). Chronic pain is defined as persistent or recurrent pain lasting more than 3 months (Treede et al., 2015). In Denmark, 20% of the general population suffers from chronic pain (Sjøgren et al., 2009). Although different clinically recommended treatment strategies can be applied in management of chronic pain, some patients may not experience adequate relief. Moreover, conventional analgesics may cause various adverse reactions, such as headache, dizziness, confusion, and constipation, and thus contribute to daily functional impairment and reduced quality of life (QoL) (Finnerup et al., 2015; Harker et al., 2012).

In this context, cannabis and cannabinoids are considered supplementary or alternative therapeutic regimens to conventional pain‐relieving treatment (Häuser, Finn, et al., 2018). The cannabis regimens contain a broad spectrum of different cannabinoids, mainly including delta‐9‐tetrahydrocannabinol (THC) and cannabidiol (CBD), and other plant elements such as terpenoids and flavonoids, whereas the cannabinoid regimens contains predominantly THC, CBD and THC/CBD, and occasionally minimal quantities of other plant‐derived substances (Häuser, Finn, et al., 2018). The theoretical explanation of a potential analgesic effect of exocannabinoids has been presented in the literature with reference to the endocannabinoid system (Howlett & Abood, 2017; Zou & Kumar, 2018; Hillard, 2015). However, reviews and meta‐analysis have reached conflicting conclusions of evidence being either inconsistent, not to be documented, low, moderate or, substantial regarding effectiveness of cannabis as medicine for relief of chronic pain in adults (Aviram et al., 2017; Bialas et al., 2022; Fisher et al., 2021; Häuser, Petzke, & Fitzcharles, 2018; McDonagh et al., 2022; National Academies of Sciences et al., 2017; Petzke et al., 2022; Sainsbury et al., 2021; Wang et al., 2021). The International Association for Study of Pain (IASP) has concluded that evidence was lacking to either support or refuse a potential pain‐relieving effect of cannabis as medicine as current randomized controlled trials (RCTs) were of low or very low quality (Fisher et al., 2021).

Assessment and establishment of clinical evidence requires access to results from RCTs, predominantly. However, observational studies, including cohort and case‐series studies, may also contribute with important data in assessment of evidence (Mariani & Pêgo‐Fernandes, 2014). Moreover, observational studies may provide vital information to serve as guidance when planning and executing high quality RCTs. Different observational studies on the effectiveness of cannabis as medicine on chronic pain have been conducted, hence contributing to the evidence pyramid. The majority of observational studies have explored medicinal cannabis in a chronic pain context (Aviram et al., 2021; Benedict et al., 2022; Boehnke et al., 2016; Fanelli et al., 2017; Haroutounian et al., 2016; Meng et al., 2021; Poli et al., 2018) as opposed to cannabinoids in a few studies only (Kawka et al., 2021; Ueberall et al., 2019, 2022).

In January 2018, a four‐year pilot programme was initiated in Denmark enabling patients to access medicinal cannabis by a prescription from a physician (Danish Medicines Agency). Even though the pilot program primarily aimed to assess medicinal cannabis products, the availability of these products in general failed during the programme due to technical complications with authorization of the submitted products by the Danish Medicines Agency. Consequently, majority of prescriptions in the pilot programme have so far been related to therapy with cannabinoids, either as biologically active constituents of cannabis, or synthetic compounds. The aim of this study was therefore to elucidate tolerability and effectiveness of oral cannabinoid therapy among patients with treatment refractory chronic pain (TRCP) during the initial period of the Danish pilot program.

## 2 METHODS

This retrospective real‐world study was conducted between August 2018 and February 2021 at the North Denmark Regional Hospital in collaboration with a Danish pain clinic.

The indication for oral cannabinoid therapy was TRCP for the patients included as study population. The definition of TRCP is pain lasting more than 3 months with insufficient pain‐relieving effectiveness or intolerable adverse events (AEs) of conventional analgesic regimens. Patients with incurable cancer and chronic cancer‐related pain were also included in the study although this group did not necessarily fulfil the definition of TRCP in relation to history of pain and conventional pain‐ relieving treatment. Common clinical guidelines for conventional analgesic regimens for treatment of chronic pain in Denmark include opioids as primary analgesic, and secondary analgesic including tricyclic antidepressants, antiepileptic drugs and serotonin and noradrenaline reuptake inhibitors. Two treatment scenarios were applied in the pain clinic in conjunction with initiation of oral cannabinoid therapy, either prescribed to a patient with a history of TRCP either as add‐on therapy to a current conventional pain‐relieving regimen upon the baseline consultation or as monotherapy if the patient was not receiving any conventional analgesics. Moreover, for the first group of patients, no changes were made to the current conventional regimen at the baseline consultation unless a patient reported intolerable adverse events to a conventional analgesic. Then, this analgesic was either decreased in dosage or discontinued.

**FIGURE 1:**
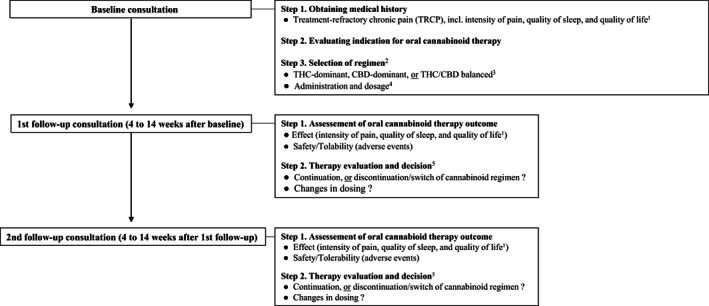
Consultation steps and oral cannabinoid therapy algorithm 1. *Pain intensity, quality of sleep, and quality of life measured by use of the numeric rating scale (NRS) ranging from 0 to 10*. 2. *Different pain characteristics and related symptoms are included when selecting a CBM regimen: THC‐dominant (neuropathic pain, nausea/vomiting, and insomnia), CBD‐dominant (inflammatory pain, anxiety, and muscle spasms), and THC+CBD balanced (neuropathic pain‐related, centralized pain, and insomnia)* 3. *THC (tetrahydrocannabinol); CBD (cannabidiol).* 4. *CBM therapy has a wide therapeutic range of dosing and is highly individual from patient to patient. Dosing follows the principles of “start low‐go slow” and patients‐determined self‐titrating. The following dosing criteria are applied in administration of cannabis‐based medicine as oil or capsule: THC‐dominant (1‐2.5 mg once a day and increase every third day with 1‐2.5 mg until effect, and up till 25 mg/day in 3 doses), CBDdominant (10 mg once a day and increase every third day with 10 mg up to 50 mg/day in 3 doses. For anti‐inflammatory effect up to 5 mg/kg/day), and THC+CBD balanced (same criteria as for THC‐dominant regimen).* 5. *Therapy evaluation and decision is based upon patient‐reported effect and adverse events, e.g. discontinuation, switch, or increasing dosing of current CBM regimen in case of inadequate pain‐relieving effect OR discontinuation, switch, pausing, or decreasing dosing of current CBM regimen in case of intolerable adverse effects.*

Patients were included in this study if the following inclusion criteria were fulfilled (Figure 1): being issued an oral cannabinoid product prescription at the Danish pain clinic from January 1st 2018 to December 31st 2018, a history of TRCP and an established diagnosis related to chronic pain, aged ≥18 years. Patients were excluded if the follow‐up consultation was not performed within 4–14 weeks from baseline to first follow‐up (F/U1), the oral cannabinoid regimen at F/U1 was not identical to that at baseline or an event had occurred in the follow‐up period having an impact on the level of pain perception reported at baseline for example, a medical/surgical procedure or an accident. Moreover, the same principles of exclusion, in addition to discontinuation of cannabinoid therapy, were also applied to second follow‐up (F/U2) (Figure 1).

Data from medical records were registered on diagnosis, pain intensity, quality of sleep, QoL, treatment‐related AEs, and cannabinoid therapy specifications. Diagnoses were presented in accordance to International Classification of Diseases version 2010 (ICD‐10) (World Health Organization, n.d.). Patient demographics and clinical data were registered and managed using the Research Electronic Data Capture (REDCap) hosted at the North Denmark Regional Hospital. REDCap is a secure, web‐based application designed to support data capture for research studies (Harris et al., 2009).

### 2.1 Oral cannabinoid regimens

Patients were prescribed purified cannabinoid products in the oral forms of sublingual oil or capsules containing (i) purified THC 0.83 mg/drop or 2.5 mg/capsule as monotherapy (THC), (ii) CBD 1.67 mg/drop, 2.86 mg/drop or 10 mg/capsule as monotherapy (CBD) or (iii) a capsule combination product with purified THC 2.5 mg and purified CBD 5 mg as combination therapy (THC/CBD). Some patients were prescribed an oral regimen containing a purified THC product together with a purified CBD product, which was then also registered as THC/CBD. The prescribed oral cannabinoid products are manufactured and controlled at Glostrup Pharmacy (Copenhagen, Denmark) according to the European Union Good manufacturing practices (EU GMP). The purified THC and CBD ingredients in the prescribed oral cannabinoid products are manufactured, controlled, and supplied to Glostrup Pharmacy by EU GMP‐approved suppliers in Europe. The treatment outcomes for the three regimens (THC, CBD, and THC/CBD, respectively) are presented regardless of route of administration and dosage.

### 2.2 Study outcomes

**FIGURE 2:**
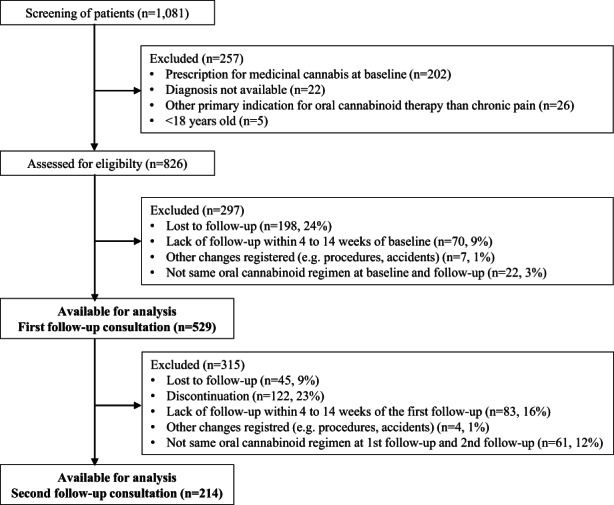
Flowchart of study participants

Figure 2 provides an overview of the baseline and follow‐up consultation steps in relation to the prescribed oral cannabinoid regimens. The decision for which of the three regimens (THC, CBD, and THC/CBD, respectively) was made upon reported treatment refractory pain as main indication for oral cannabinoid therapy, but secondary complaints, such as sleep disturbances, anxiety, nausea and muscle spasms, were taken into consideration (Figure 2). Safety and effectiveness outcomes were based on data from the baseline consultation and F/U1 between 4 and 14 weeks after oral cannabinoid therapy had been initiated in patients at baseline. Outcomes from F/U2 were also registered if the consultation likewise had also been undertaken within a 4–14 weeks period after F/U1. The reasons for the defined time range were that it was expected to take a minimum of 4 weeks to stabilize dosage and a maximum of 14 weeks for a potential effect to occur at an adequate level to be reported by a patient. Safety and effectiveness outcomes were registered at each consultation (Figure 1).

### 2.3 Safety

The patients were at the follow‐up consultations in the pain clinic asked if any AEs had occurred during course of treatment, and if so, status of potential causality to oral cannabinoid therapy was assessed on discretion of the attending physician. If a plausible relation was suspected or could not be ruled out, the treatment‐related AEs was registered in the patient medical record. After data collection AEs were divided in groups based upon Common Terminology Criteria for Adverse Events (CTCAE) 5.0 (National Cancer Institute, 2017). However, the grade of seriousness of the AEs were not available in the medical records.

### 2.4 Effectiveness

The primary effectiveness outcome was difference in mean pain intensity between baseline and follow‐up consultations. To measure patient‐reported pain intensity, a numeric rating scale (NRS) ranging from 0 to 10 (0 = no pain, 10 = worst pain) was used. Patients were asked at baseline and follow‐up to state the perceived level of pain intensity within the last 3 days.

Secondary effectiveness outcomes were measured as semi‐structured differences in quality of sleep and QoL between baseline and follow‐up consultations using a simple non‐validated approach. Patients were asked by the physician how they were feeling lately without precise time limitation. The outcomes were assessed as patient‐reported outcome (PRO) items at each consultation using the three following response categories: (1) improved, (2) no changes, or (3) worsened.

### 2.5 Data quality

One and the same person (KLH) entered data in REDCap as a measure against risk of inter‐observer errors. A quality control of data was completed in 54 of the 1081 screened patients (5%), who were randomly selected to assess intra‐observer reliability. KLH inspected if the entered data in REDCap was consistent with data in patient medical records. Entry errors were divided into mild, moderate, and severe, respectively. Mild errors were of no importance for results, moderate errors were of some importance for results, but they were corrected for all patients during data management and severe errors were of great importance for results. Severe errors were detected in 3% of the patients included in the quality control analysis. It was concluded that the entered data were of high reliability and no further quality control was needed.

### 2.6 Ethics and data protection

This study did not require ethical approval from the Danish National Committee on Health Research Ethics. Disclosure of data from patients' medical records from the Danish pain clinic to the North Denmark Regional Hospital was approved by the Danish Patient Safety Authority (3–3013‐2588/1), wherefore signed informed consent from the patients was not required. The study was approved by the Danish Data Protection Agency (2018–102).

### 2.7 Statistics

Data were examined by descriptive analysis and are presented as percentage, and some parametric data are presented as mean ± standard deviation (SD) and median interquartile range (IQR) if non‐normal distributed. Each analysis was performed for each of the three regimens (THC, CBD, and THC/CBD, respectively) and a total group. Primary effectiveness outcome and secondary PRO were both analysed by comparing data at baseline and follow‐up. A reduction in pain intensity ≥30% between baseline and follow‐up was considered clinical relevant (Dworkin et al., 2005). Gender, diagnosis, AEs, percentage change in paired mean NRS and changes in PRO in quality of sleep and QoL were analysed by Chi^2^. Normal distributed data regarding age, body mass index (BMI) and difference in NRS between the oral cannabinoid regimens were analysed by one‐way ANOVA. Nonparametric data regarding number of days from baseline to follow‐up were analysed by Kruskal‐Wallis method. Moreover, Tukey Studentized Range Test was applied for additional post hoc testing. Comparison of changes in NRS at baseline and follow‐up within the three individual oral cannabinoid regimens was analysed by paired t‐test as data was normal distributed. A *p*‐value<0.05 was defined as statistically significant. Missing data was handled by pairwise deletion. *Per‐protocol* data analyses were supplemented with *intention‐to‐treat* data analyses. Data were analysed using SAS Enterprise Guide 7.1 and 8.3.

## 3 RESULTS

Of 826 eligible patients, 529 (64%) were included in the final analysis of F/U1 data and among those 214 patients (40%) were included for analysis of F/U2 data (Figure 2). The median interval between the baseline consultation and F/U1 was 56 (42–65) days in comparison to 126 (105–147) days between the baseline consultation and F/U2. In general, longer follow‐up intervals were observed for patients in the CBD and THC/CBD groups compared with patients in the THC group (*p* = 0.0017) (Table 1a). No significant difference of importance was found when comparing included patients within the range of 4–14 weeks and excluded patients within <4 and >14 weeks (Table S1). Also, no significant difference in demographic, clinical characteristics, and oral cannabinoid regimens were found between the patient groups attending baseline, F/U1 and F/U2 except from a significant higher number of patients with malignant disease (*p* = 0.0098) (Table S2).

**TABLE 1:** Demographic and clinical characteristics of patients with treatment refractory chronic pain receiving oral cannabinoid therapy (*N* = 529)

	THC	CBD	THC/CBD	*p*‐value	Total
	*n* = 284	*n* = 198	*n* = 47		*N* = 529
A. Characteristics					
Gender, *n* (%)					
Female	180 (63)	154 (78)	34 (72)	0.0030	368 (70)
Male	104 (37)	44 (22)	13 (28)		161 (30)
Age					
Mean years ± SD	61 ±15.2	59 ±16.9	51 ±12.5	<0.0001	60 ±15.9
BMI					
Mean ± SD	26.4 ±5.9	25.6 ±5.5	24.6 ±0.9	0.0961	25.9 ±5.7
Days from baseline to follow‐up					
Median (IQR)	49 (40–63)	57 (44–68)	63 (40**–**77)	0.0017	56 (42–65)
Range	28–98	28–98	28–98		28–98
Dose (mg)					
Median per day (IQR)	7.5 (7.5–14.9)	33.4 (33.4–33.4)	7.1 (3.8–15.0) +	—	—
			31.7 (20.9–33.4)		
Range	0.8–24.9	3.3–125.3	0.8–40 + 1.7–50.1	—	—
Missing	32	17	5 + 7		
B. Diagnostic categories, *n* (%)					
Diseases of the musculoskeletal system and connective tissue (DM00‐DM94)^a^	51 (18)	64 (32)	11 (23)	0.0013	126 (24)
Injury, poisoning and certain other consequences of external causes (DS00‐ DT98)^b^	77 (27)	33 (17)	9 (19)	0.0220	119 (23)
Diseases of the nervous system (DG00‐ DG99)^c^	35 (12)	20 (10)	3 (6)	0.4275	58 (11)
Malignant neoplasms (DC00‐DC97) and cancer‐related medical care inducing neuropathic pain^d^	32 (11)	5 (3)	9 (19)	0.0001	46 (9)
Other diagnoses^e^	20 (7)	12 (6)	2 (4)	0.7440	34 (6)
Multiple diagnoses^f^	69 (24)	64 (32)	13 (28)	0.1524	146 (28)

*Note*: THC (Tetrahydrocannabinol); CBD (Cannabidiol); SD (Standard deviation), BMI (Body mass index), IQR (Interquartile range). Statistics: Chi^2^ (Gender; Diagnostic categories), One‐way ANOVA (Age; BMI), Kruskal–Wallis (Days from baseline to follow‐up).

^a^ Fibromyalgia *n* = 30, arthrosis *n* = 26, rheumatoid arthritis *n* = 18, degenerative disk disease *n* = 12, spinal stenosis *n* = 9, scoliosis *n* = 7, herniated disc *n* = 6, other musculoskeletal diseases *n* = 17.

^b^ Post‐surgery *n* = 80, post‐injury=35, other external causes *n* = 5.

^c^ Neuropathies *n* = 29, headache *n* = 11, systemic atrophies primarily affecting the central nervous system for example, Parkinson *n* = 6, other neurological diseases *n* = 12.

^d^ Breast cancer *n* = 12, Cancer in digestive organs *n* = 10, cancer presumed to be primary, of lymphoid, haematopoietic and related tissue *n* = 6, respiratory and cancer in male genital organs *n* = 5, other malignant neoplasms *n* = 13. Cancer with metastases *n* = 17 (37%).

^e^ Other diagnoses cover “Congenital malformations, deformations and chromosomal abnormalities (DQ00‐99)” for example, Ehlers‐Danlos, “Symptoms, signs and abnormal clinical and laboratory findings, not elsewhere classified (DR00‐DR99)” for example, burning mouth syndrome, “Endocrine, nutritional and metabolic diseases (DE00‐DE90) for example, Fabry disease, “Diseases of the blood and blood‐forming organs and certain disorders involving the immune mechanism (DD50‐DD89)” for example, MBL deficiency, “Certain infectious and parasitic diseases (DA00‐DB99)” for example, HIV, “Diseases of the digestive system (DK00‐DK93)” for example, Crohns disease, “Diseases of the genitourinary system (DN00‐DN99) for example, endometriosis.

^f^ Patients registered with more than one diagnosis as the reason fororal cannabinoid therapy.

### 3.1 Demographic and clinical characteristics

The majority of the 529 patients prescribed oral cannabinoid products in the pain clinic were females (70%). More females were prescribed an oral cannabinoid regimen (*p* = 0.003). The mean age of the Overall population was 60 ± 15.9 years, and mean BMI was 25.9 ± 5.7 (Table 1a). Among the 529 patients 46 (9%) were registered with cancer‐related pain.

The distribution of the three oral cannabinoid regimens were as follows: THC (*n* = 284, 54%), CBD (*n* = 198, 37%) and THC/CBD (*n* = 47, 9%) (Table 1a). The median dose of THC therapy was 7.9 mg per day at F/U1 and 10.6 mg per day at F/U2. The median dose of CBD therapy was 35 mg per day at both F/U1 and F/U2. The median dose of THC/CBD therapy was 7.9 + 33 mg per day at F/U1 and 13.2 + 29 mg per day at F/U2. The highest proportion of male patients was observed in the THC group in comparison to the two CBD containing regimen groups, whereas it was opposite for female patients. The patients in the THC/CBD group were younger with a mean age of 51 ± 12.5 years (*p* < 0.0001). A total of 146 patients (28%) treated with oral cannabinoid products had been registered with more than one diagnosis associated with perception of chronic pain and by which oral cannabinoid therapy was considered an option (Table 1b). The most common diagnostic categories were related to diseases of the musculoskeletal system (24%), and injury, poisoning and certain other consequences of external causes (23%). Preferred oral cannabinoid regimen depended on diagnostic group regarding musculoskeletal system (CBD; *p* = 0.0013), injury, poisoning and certain other consequences of external causes (THC; *p* = 0.0220) and malignant neoplasm (THC/CBD; *p* = 0.0001).

### 3.2 Safety

A total of 42% patients reported one or more AEs during oral cannabinoid therapy at F/U1 (Table 2a) and 34% reported a least one AE at F/U2 (Table 3a). At F/U1, AEs were more often reported in oral cannabinoid therapy regimens containing THC (*p* < 0.0001), while no significant difference was observed at F/U2. Complaints related to the gastrointestinal system (F/U1:17% and F/U2: 13%), the nervous system (F/U1: 14% and F/U2: 11%) and general disorders and administration site conditions (F/U1: 14% and F/U2: 9%) were the most predominant categories of AEs. A detailed overview of AEs is presented in Table S3 (F/U1) and Table S4 (F/U2), where most frequently reported specific AEs were fatigue (F/U1:13% and F/U2: 9%) and dry mouth (F/U1: 9% and F/U2: 6%). At F/U1, gastrointestinal and general AEs were more often reported by patients treated with THC, either as monotherapy or in combination with CBD (*p* = 0.0011 and *p* = 0.0245, respectively). AEs in the nervous system were more frequently observed in patients treated with THC monotherapy (*p* < 0.0001). No difference between oral cannabinoid regimens and reported AE categories were observed at F/U2. One patient (<1%) developed hallucinations and was hospitalized due to intake of a higher THC dosage than instructed by the attending physician in the pain clinic. Treatment with THC was then discontinued.

**TABLE 2:** Overview of adverse events and effectiveness reported in accordance with different oral cannabinoid regimens at first follow‐up consultation (*N* = 529)

	THC *N* = 284	CBD *N* = 198	THC/CBD *N* = 47	*p*‐value	Total *N* = 529
(A) Adverse events, *n* (%)					
One or more adverse reactions	145 (51)	59 (30)	19 (40)	<0.0001	223 (42)
Gastrointestinal disorders	64 (23)	19 (10)	8 (17)	0.0011	91 (17)
Nervous system disorders	58 (21)	13 (7)	5 (11)	<0.0001	76 (14)
General disorders and administration site conditions	49 (17)	17 (9)	7 (15)	0.0245	73 (14)
Psychiatric disorder	17 (6)^a^	7 (4)	0	0.1307	24 (5)
Vascular disorders	5 (2)	0	1 (2)	NA	6 (1)
Musculoskeletal disorders	1 (<1)	4 (2)	1 (2)	NA	6 (1)
Skin and subcutaneous tissue disorders	1 (<1)	2 (1)	0	NA	3 (1)
Eye disorders	1 (<1)	1 (1)	0	NA	2 (<1)
Respiratory disorders	0	1 (1)	0	NA	1 (<1)
Cardiac disorders	0	1 (1)	0	NA	1 (<1)
Sensory disorders	0	0	1 (2)	NA	1 (<1)
Other disorders	6 (2)	2 (1)	2 (4)	NA	10 (2)
Missing, *n*	1	0	0		1
(B) NRS, collectively mean of means ± SD					
Baseline consultation	7.3 ± 1.6	6.8 ± 1.6	6.7 ± 1.9	0.0052	7.0 ± 1.7
Follow‐up consultation	5.8 ± 2.3	5.6 ± 2.4	4.6 ± 2.5	0.0200	5.6 ± 2.4
*p*‐value	<0.0001	<0.0001	<0.0001	—	<0.0001
Mean reduction NRS from baseline to follow‐up	1.5 ± 2.1	1.2 ± 2.2	1.9 ± 2.5	0.0662	1.4 ± 2.2
Missing, *n*	59	32	7		98
(C) Percentage change in paired mean NRS, *n* (%)					
Increase NRS	34 (15)	34 (21)	5 (13)	0.2759	73 (17)
No change NRS	37 (16)	30 (18)	6 (15)	0.8618	73 (17)
Reduction NRS >0–<30%	86 (38)	50 (30)	9 (23)	0.0720	145 (34)
Reduction NRS ≥30%–<50%	27 (12)	26 (16)	10 (25)	0.0891	63 (15)
Reduction NRS ≥50%	41 (18)	26 (16)	10 (25)	0.3761	77 (18)
Missing, *n*	59	32	7		98
(D) Patient‐reported quality outcomes					
Quality of sleep					
Improved	133 (53)	98 (55)	26 (63)	0.4579	257 (55)
No change	115 (46)	71 (40)	14 (34)	0.2471	200 (43)
Worsened	3 (1)	9 (5)	1 (2)	0.0553	13 (3)
Missing, *n*	33	20	6		59
Quality of life					
Improved	132 (56)	88 (53)	29 (78)	0.0175	249 (57)
No change	95 (40)	76 (46)	8 (22)	0.0248	179 (41)
Worsened	10 (4)	2 (1)	0	0.1066	12 (3)
Missing, *n*	47	32	10		89

*Note*: Statistics: Chi^2^ (Adverse events, Percentage change in NRS, Patient‐reported quality outcomes), One‐way ANOVA (NRS difference between CBM regimens), Paired *t*‐test (NRS difference between baseline and follow‐up).

Abbreviations: CBD, Cannabidiol; NRS, Numeric rating scale; SD, Standard deviation; THC, Tetrahydrocannabinol.

^a^ One patient (0.2%) developed hallucinations following intake of THC. The patient did not comply with the recommended dosage guideline.

**TABLE 3:** Overview of adverse events and effectiveness reported in accordance with different oral cannabinoid regimens at second follow‐up consultation (*N* = 214)

	THC *N* = 110	CBD *N* = 82	THC/CBD *N* = 22	*p*‐value	Total *N* = 214
A. Adverse events, *n* (%)					
One or more adverse reactions	41 (37)	22 (27)	9 (41)	0.2021	72 (34)
Gastrointestinal disorders	16 (15)	9 (11)	2 (9)	0.5564	27 (13)
Nervous system disorders	16 (15)	4 (5)	3 (14)	0.0912	23 (11)
General disorders and administration site conditions	14 (13)	4 (5)	2 (8)	0.1810	20 (9)
Psychiatric disorder	4 (4)	2 (2)	0	0.8842	6 (3)
Vascular disorders	2 (2)	0	0	NA	2 (1)
Musculoskeletal disorders	1 (1)	4 (5)	0	NA	5 (2)
Skin and subcutaneous tissue disorders	0	1 (1)	0	NA	1 (<1)
Eye disorders	0	0	1 (5)	NA	1 (<1)
Respiratory disorders	1 (1)	1 (1)	1 (5)	NA	3 (1)
Cardiac disorders	1 (1)	0	0	NA	1 (<1)
Sensory disorders	0	0	0	NA	0
Other disorders	1 (1)	0	1 (4)	NA	2 (1)
Missing, *n*	0	1	0		2
B. NRS, collectively mean of means ± SD					
Baseline consultation	7.2 ± 1.8	6.8 ± 1.7	6.8 ± 2.0	0.4211	7.0 ± 1.8
Follow‐up consultation	5.4 ± 2.4	5.0 ± 2.8	4.6 ± 2.4	0.3860	5.1 ± 2.5
*p*‐value	<0.0001	<0.0001	0.0006	—	<0.0001
Mean reduction NRS from baseline to follow‐up	1.8 ± 2.3	1.8 ± 2.6	2.4 ± 2.4	0.5839	1.8 ± 2.4
Missing, *n*	18	17	4		39
C. Percentage change in paired mean NRS, *n* (%)					
Increase NRS	12 (13)	13 (20)	2 (11)	0.4276	27 (15)
No change NRS	11 (12)	9 (14)	1 (6)	0.6320	21 (12)
Reduction NRS >0 ‐ <30%	31 (34)	15 (23)	2 (11)	0.0889	48 (27)
Reduction NRS ≥30%‐ < 50%	20 (22)	9 (14)	6 (33)	0.1563	35 (20)
Reduction NRS ≥50%	18 (20)	19 (29)	7 (39)	0.1419	44 (25)
Missing, *n*	18	17	4		39
D. Patient‐reported quality outcomes, *n* (%)					
Quality of sleep					
Improved	50 (49)	31 (44)	13 (77)	0.0511	94 (49)
No change	45 (44)	37 (52)	3 (18)	0.0359	85 (45)
Worsened	8 (8)	3 (4)	1 (6)	0.6375	12 (6)
Missing, n	7	11	5		23
Quality of life					
Improved	48 (51)	35 (60)	10 (71)	0.2558	93 (56)
No change	38 (40)	21 (36)	2 (14)	0.1659	61 (37)
Worsened	8 (9)	2 (4)	2 (14)	0.2856	12 (7)
Missing, *n*	16	24	8		48

*Note*: Statistics: Chi^2^ (Adverse events, Percentage change in NRS, Patient‐reported quality outcomes), One‐way ANOVA (NRS difference between oral cannabinoid regimens), Paired *t*‐test (NRS difference between baseline and follow‐up).

Abbreviations: CBD, Cannabidiol; NRS, Numeric rating scale; SD, Standard deviation; THC, Tetrahydrocannabinol.

### 3.3 Effectiveness

Comparison of mean pain intensity on NRS at baseline versus at F/U1 and F/U2 is presented in Table 2b and Table 3b, respectively. A total of 10–20% of data were missing. In overall, the patients reported a mean reduction of 1.4 at F/U1 and 1.8 at F/U2 on NRS (*p* < 0.0001). The THC group had a mean reduction in pain intensity of 1.5 at F/U1 and 1.8 at F/U2 in comparison to 1.2 at F/U1 and 1.8 at F/U2 in the CBD group, and 1.9 at F/U1 and 2.4 at F/U2 in the THC/CBD group. The reduction of mean NRS at F/U1 and F/U2 was significant (*p* < 0.0001 and *p* = 0.0006, respectively) for all three oral cannabinoid regimens.

Table 2C and Table 3C shows the paired mean percentage differences between baseline and follow‐ up (F/U1 and F/U2, respectively) in mean NRS. A total of 73 patients (17%) experienced an increase in pain intensity at F/U1 and 27 patients (15%) at F/U2. At F/U1, the same number of patients (*n* = 73, 17%) experienced no changes in pain intensity in comparison to 21 patients (12%) at F/U2. A total of 285 patients (66%) experienced a reduction in NRS at F/U1 and 129 patients (73%) at F/U2. Per‐protocol analysis revealed that one in three patients (32%) experienced a clinically relevant reduction in pain intensity of at least 30% in NRS at F/U1, while almost half (*n* = 79, 45%) had a reduction in NRS of at least 30% at F/U2. By per‐protocol analysis, a pain reduction of ≥30% was also observed in 30% (F/U1) and 41% (F/U2) of patients who were prescribed THC. The figures were 31% (F/U1) and 43% (F/U2) for patients treated with CBD as opposed to 50% (F/U1) and 72% (F/U2) treated with THC + CBD. Patients prescribed THC/CBD were significantly more like to obtain a ≥ 30% pain reduction than THC and CBD as monotherapy (F/U1: *p* = 0.0446 and F/U2: *p* = 0.05). The number of eligible patients intended for oral cannabinoid therapy was 826 and taken this figure into consideration. Hence, intention‐to‐treat analysis revealed that 17% at F/U1 and 10% at F/U2 of the baseline population reported a clinically relevant reduction of ≥30% in pain intensity.

A significant higher number of patients with chronic cancer‐related pain compared with non‐cancer‐ related pain reported ≥50% reduction in NRS (42% vs. 16%, *p* = 0.0003) (Table S5). Figures from intention‐to‐treat analysis were 14% for cancer‐related pain and 9% for non‐cancer‐related pain, respectively.

Also, a higher number of patients with chronic cancer‐related pain were prescribed THC, either as monotherapy or in combination with CBD (*p* = 0.05 and *p* = 0.006, respectively), while patients with non‐cancer‐related pain were more likely to be treated with CBD monotherapy (*p* = 0.0003). Differences in PRO, including changes in quality of sleep and QoL after initiation of oral cannabinoid therapy are presented in Table 2D (F/U1) and Table 3D (F/U2), respectively. A total of 257 (55%) at F/U1 and 94 (49%) at F/U2 reported improvement in sleep and 249 (57%) at F/U1 and 93 (56%) at F/U2 reported improvement in QoL by per‐protocol analysis. Of notice, improvements in QoL were more commonly reported by patients treated with a THC/CBD as opposed to patients treated with a mono‐cannabinoid regimen (*p* = 0.0175) at F/U1. This tendency was also observed at F/U2 regarding quality of sleep (*p* = 0.05). For intention‐to‐treat, 30% of eligible patients at F/U1 and 11% at F/U2 reported uniformly improvement in both sleep and QoL.

### 3.4 Missing follow‐up data

As presented earlier, F/U1 data were not available in 297 (36%) of the 826 patients having attended a baseline consultation (Figure 2). A total of 198 patients (24%) were registered as lost to follow‐up. These patients had been prescribed an oral cannabinoid regimen with the following distribution: THC (*n* = 109, 55%), CBD (*n* = 74, 37%), and THC/CBD (*n* = 15, 8%). In this group, 26 patients (13%) died before follow‐up, 24 with a cancer diagnosis. After F/U1, 45 patients (9%) were registered as lost to follow‐up, and an additional 122 patients (23%) discontinued oral cannabinoid therapy (Figure 2) mostly, for unknown reasons (*n* = 46, 38%). As known reasons were registered no perceived effect (*n* = 36, 30%), AEs (*n* = 15, 12%), death (*n* = 13, 11%), insufficient funds (*n* = 9, 7%) and other reasons 279 (*n* = 6, 5%).

## 4 DISCUSSION

This retrospective study of a large population of patients with TRCP, and chronic cancer‐related pain, presents safety and effectiveness data regarding the use of oral cannabinoid therapy in Danish pain clinic setting. With respect to safety, 42% of patients receiving oral cannabinoid therapy at F/U1 and 34% at F/U2 reported one AE or more. The reported prevalences were higher than presented in an open‐label real‐world study in which 19% of patients with chronic pain receiving oral cannabinoid therapy using THC/CBD oromucosal spray reported at least one treatment‐emergent AE after 12 weeks (Ueberall et al., 2019) in comparison to 47% for THC monotherapy more recently also reported from the German Pain e‐Registry group (Ueberall et al., 2022). The most frequently reported AEs in our study were related to gastrointestinal disorders (e.g. dry mouth), in addition to general disorders and administration site conditions (e.g. fatigue). The AEs were predominantly occurring in patients receiving THC monotherapy regimen as opposed to CBD containing regimen, in particular nervous system disorders (e.g. dizziness and headache). This observation supports the current assumption that CBD in combination therapy with THC may have an alleviating effect on potential AEs caused by THC monotherapy (MacCallum & Russo, 2018).

The most common AEs in our study are similar to what have been reported earlier among chronic pain patients treated with oral cannabinoids (Kawka et al., 2021). Our study found in general AEs to be mild to moderate in intensity. However, one patient (0.2%) experienced a serious AE requiring hospitalization due to hallucinations following non‐compliant increased THC dosing by the patient. Of further notice, a substantial proportion of patients in our study had no available follow‐up data. Consequently, the number of AEs could potentially be higher, and of more severe nature. Therefore, conclusion about safety in this study should be made with caution.

With respect to effectiveness, per‐protocol analysis revealed that 32% of all patients receiving oral cannabinoid therapy at F/U1 and 45% at F/U2 experienced a pain reduction of 30% or more when comparing reported mean NRS pain intensity in the past 3 days at baseline versus follow‐up consultations. However, the figures were 17% and 10%, respectively, in intention‐to‐treat analysis. Of interest, a higher proportion of patients treated with THC/CBD achieved ≥30% pain reduction compared to THC and CBD as monotherapy as earlier reported. The same tendency was revealed in two recent reviews (McDonagh et al., 2022; Sainsbury et al., 2021). Moreover, among patients at F/U1 at F/U2 18% and 25%, respectively, experienced a pain reduction of ≥50% in comparison to 21% as earlier reported (Bialas et al., 2022). However, the figures are lower than the reported by German Pain e‐Registry group where 47% and 68% for patients being prescribed THC and THC/CBD, respectively (Ueberall et al., 2019, 2022). Also, in the latter studies, median doses were 15.0 mg (THC) and 18.9 + 17.8 mg (THC/CBD) per day (Ueberall et al., 2019, 2022) while in our study patients in general were prescribed lower doses: 7.9 mg at F/U1 and 10.6 mg per day at F/U2 for THC monotherapy, and 7.9 + 33 mg per day at F/U1 and 13.2 + 29 mg per day at F/U2 for THC/CBD. Hence, comparison of the findings from the presented studies suggests that dosing of THC to the patient with chronic pain is not only positively correlated to reported effectiveness but also to poor tolerability as a consequence.

Of interest, our study revealed that a significant higher proportion of patients with chronic cancer‐related pain reported ≥50% reduction in NRS in comparison to patients with non‐cancer‐related pain (42% versus 16%). However, the findings are related per‐protocol analysis, whereas the intention‐to‐ treat analysis could not demonstrate any difference of importance (14% versus 9%, respectively). Moreover, the former group was also more frequently treated with THC containing regimens, which may have a potential confounding effect. Of notice, pain is not the only complaint typically reported by this group of patients addressed. The patients may also have other complaints, including sleep disturbances, anxiety, loss of appetite, nausea, muscle spasms and so forth and for which THC may provide additional benefits, which may then have a positive impact on pain perception.

The lack of follow‐up for major proportion of patients in this study could be caused by insufficient pain‐relieving effect of oral cannabinoid therapy, occurrence of AEs or both. In the intention‐to‐treat data analyses, NRS pain reduction of ≥30% was confirmed in 17% at F/U1 and 10% at F/U2 of the 826 study eligible patients attending the baseline consultation, equal to one out of six and one out of 10 patients, respectively. When interpreting this effectiveness outcome, one should take into consideration that the group of patients in this study were characterized as potential difficult‐to‐treat patients with chronic pain. In that perspective oral cannabinoid therapy could be perceived as a justified approach for management of chronic pain in particular for a subgroup of patients failing conventional treatment or experiencing intolerable AEs.

Of interest, 17% (F/U1) and 15% (F/U2) of the patient cohort reported an increase in pain intensity by NRS at follow‐up. The findings indicate that some patients have not achieved a desirable effect of oral cannabinoid therapy, or simply that some patients' medical condition deteriorates from baseline to follow‐up. The German Pain e‐Registry group investigating effectiveness and tolerability of THC/CBD found that patients with nociceptive pain in general reported a deterioration of pain using a visual analogue scale (VAS) after 12 weeks of treatment. In comparison, patients with neuropathic pain or a mix of neuropathic and nociceptive pain experienced an improvement in pain by VAS (Ueberall et al., 2019). The patients in our study were not categorized according to type of chronic pain, and therefore it was not possible to explore further into differences in response to oral cannabinoid therapy in that context. In matter of fact, the study population was rather heterogeneously composed which was a challenge to the overall data analysis and to the interpretation of the study results, taking the different diagnostic groups and pain phenotypes into account.

Poor quality of sleep is frequently reported by patients with chronic pain (Gerhart et al., 2017). In our study population, a total of 55% at F/U1 and 49% at F/U2 reported improvement in quality of sleep by per‐protocol analysis. Moreover, a beneficial outcome on QoL was reported by 57% and 56% of patients at F/U1 and F/U2, respectively, also by per‐protocol analysis. However, in intention‐to‐treat analysis figures were 30% at F/U1 and 11% at F/U2 regarding improvement in sleep and QoL, respectively. A significant higher proportion of patients reported improvement in QoL when treated with a combination of THC and CBD (78%) compared with THC or CBD as monotherapy (56% and 53%, respectively). This tendency was also observed regarding patient reported quality of sleep at F/U2, which suggests that THC and CBD in combination entails improved outcomes on this parameter as well. The findings are in close alignment with Kawka et al. and Ueberall et al., 2022, who found significant improvements regarding quality of sleep and QoL (Kawka et al., 2021; Ueberall et al., 2022). Also, a review found that medical cannabis and cannabinoids could lead to minor improvements in sleep compared to placebo in patients with both cancer and non‐cancer pain (Aminilari et al., 2022). The improvements in quality of sleep and QoL are likely secondary benefits experienced by the patients following pain reduction by oral cannabinoid therapy, as they theoretically could also lead to a higher tolerance of pain.

Our study has some major limitations, which may have different implications on the conclusions to be drawn. Firstly, conducted as an observational study there is obviously no control group in the study. Most of the patients attending the pain clinic actively searched for new pain‐relieving treatment options, which may contribute to the likelihood of analgesic placebo effect to occur. However, a review regarding placebo responses in pain syndrome, suggests that placebo effect is most significant at shorter duration (hours to days), but tend to diminish within a few weeks (Mbizvo et al., 2015).

With the relatively long follow‐up in our study (median 56 days at F/U1 and 126 days at F/U2) it is likely that the potential placebo effect is of less importance in interpretation of the effectiveness results. Second, extraction and subsequent structuring of real‐world data for analytic purpose is often challenged when using medical records as main source of data in retrospective studies. Patient data may not always be registered systematically and in a uniform way by the health care professionals in the daily clinical practice. As a result, a proportion of data are categorized as missing, which was also the case in this study in a range of 10% to 20% of patients with incomplete datasets. Thirdly, the pain clinic did not use validated questionnaires to collect PRO, which may yield less valid data and conclusion should be made with caution. Fourthly, in overall, NRS is a subjective instrument and rather sensitive for day to day, and even hour to hour variation. In future consultations, validated questionnaires will be incorporated routinely in the clinic, where the study took place. As a final limitation, patients were not anonymous when reporting outcomes at consultation with the physician which might influence their responses.

A strength of this study was that patients were treated using the same portfolio of oral cannabinoid products consistently from the same pharmacy manufacturer. Moreover, the clinical guidelines for oral cannabinoid regimens, including administration and dosing, were also applied uniformly in the pain clinic. Both elements, oral cannabinoid products and clinical guidelines, should be considered as important prerequisites in the overall process of data analysis and interpretation. Of notice, the study was conducted in a single site as opposed to multiple sites and therefore the study results should be interpreted and translated into a general practice context with some caution.

In conclusion, oral cannabinoid therapy in general appears to be safe and effective for relief of chronic pain in some patients, including a subset of patients with cancer‐related pain (9%), not responding adequately to conventional treatment regimens or experiencing intolerable AEs. Moreover, beneficial effects on sleep and QoL were reported by the patients receiving oral cannabinoid therapy, although the assessment was not performed in a validated manner. Hence, our study confirms previously reported findings related to patients with chronic pain receiving oral cannabinoid therapy and in that way the study contributes further to the evidence pyramid at the level of observational studies. The findings encourage more initiatives to be taken towards conduction of RCTs aiming at a higher level of evidence clarification. Emphasis should be made on addressing diagnosis‐specific patient groups with different pain types representing distinct pathophysiological characteristics, and possible in need of different analgesic therapy strategies.

## AUTHOR CONTRIBUTIONS

TH, KH and PL designed and conceptualized the study. TH performed the measurements and handled the subjects. KH performed data entry, statistical analysis and wrote the manuscript. All authors have contributed to the final version of the manuscript.

## FUNDING INFORMATION

The study was partially funded by Bionorica SE, and Oda and Hans Svenningsens Research Grant. The sponsors had no role in the design or reporting of this study.

## CONFLICTS OF INTEREST

KH and PL declare no conflicts of interest. TH has provided medical care to the patients in the study.

## Supporting information


Table S1.
Table S2.
Table S3.
Table S4.
Table S5.
