# Supplementary material for: Safety and effectiveness of cannabinoids to Danish patients with treatment refractory chronic pain—A retrospective observational real‐world study
Source: Eur J Pain. 2022 Dec 15;27(2):234–47. doi: 10.1002/ejp.2054 (PMC10107230; doi:10.1002/ejp.2054)
Supplement: Supplementary file 1 — Table S1. Table S2. Table S3. Table S4. Table S5. [file EJP-27-234-s001.docx]

**Supplementary**

Table S1. Demographic and clinical characteristics of patients with treatment refractory chronic pain receiving oral cannabinoid therapy (N=601)

|  | Weeks from baseline to follow-up | | |  |  |
| --- | --- | --- | --- | --- | --- |
| A. Characteristics | **<4**  **n=25** | **>14**  **n=47** | **4-14**  **n=529** | **P-value** | **Total**  **N=601** |
| Gender, n (%) |  |  |  |  |  |
| Female | 12 (48) | 33 (70) | 368 (70) | 0.0736 | 413 (69) |
| Male | 13 (52) | 14 (30) | 161 (30) |  | 188 (31) |
| Age |  |  |  |  |  |
| Mean years ±SD^d^ | 57 ±13.9 | 55 ±16.1 | 60 ±15.9 | 0.1051 | 59 ±15.8 |
| BMI |  |  |  |  |  |
| Mean ±SD | 26.5 ±5.6 | 27.1 ±5.8 | 25.9 ±5.7 | 0.3666 | 26.0 ±5.7 |
| Days from baseline to follow-up |  |  |  |  |  |
| Median (IQR) | 19 (11-23) | 126 (106-165) | 56 (42-65) |  | 56 (41-70) |
| Range | 2-27 | 99-282 | 28-98 |  | 2-282 |
| B. Diagnostic categories, n (%) |  |  |  |  |  |
| Diseases of the musculoskeletal system and connective tissue (DM00-DM94) | 2 (8) | 10 (21) | 126 (24) | 0.1774 | 138 (23) |
| Injury, poisoning and certain other consequences of external causes (DS00-DT98) | 6 (24) | 9 (19) | 119 (23) | 0.8510 | 134 (22) |
| Diseases of the nervous system (DG00-DG99) | 3 (12) | 7 (15) | 58 (11) | 0.7130 | 68 (11) |
| Malignant neoplasms (DC00-DC97) and cancer-related medical care inducing neuropathic pain | 6 (24) | 3 (6) | 46 (9) | 0.0274 | 55 (9) |
| Other diagnoses | 2 (8) | 4 (9) | 34 (6) | 0.8280 | 40 (7) |
| Multiple diagnoses | 6 (24) | 14 (30) | 146 (28) | 0.8718 | 166 (28) |
| C. Oral cannabinoid regimen prescribed at baseline, n(%) |  |  |  |  |  |
| THC | 16 (64) | 24 (51) | 284 (54) | 0.5520 | 324 (54) |
| CBD | 9 (36) | 22 (47) | 198 (37) | 0.4363 | 229 (38) |
| THC+CBD | 0 | 1 (2) | 47 (9) | 0.0843 | 48 (8) |

SD (Standard deviation), BMI (Body mass index), IQR (Interquartile range), THC (Tetrahydrocannabinol), CBD (Cannabidiol).

Statistics: Chi^2^ (Gender; Diagnostic categories, type of CBM regimen), One-way ANOVA (Age; BMI), Kruskal Wallis (Days from baseline to follow-up).

Table S2. Demographic and clinical characteristics of patients with treatment refractory chronic pain receiving oral cannabinoid therapy at baseline, first and second follow-up consultation, respectively

|  | Consultation | | |  |
| --- | --- | --- | --- | --- |
| A. Characteristics | **Baseline**  **N=826** | **First follow-up**  **N=529** | **Second follow-up**  **N=214** | **P-value** |
| Gender, n (%) |  |  |  |  |
| Female | 562 (68) | 368 (70) | 141 (66) | 0.6094 |
| Male | 264 (32) | 161 (30) | 73 (34) |  |
| Age |  |  |  |  |
| Mean years ±SD | 59 ±16.0 | 60 ±15.9 | 58 ±16.1 | 0.5757 |
| BMI |  |  |  |  |
| Mean ±SD | 25.9 ±5.6 | 25.9 ±5.7 | 26.2 ±6.0 | 0.7717 |
| Days from baseline to follow-up |  |  |  |  |
| Median (IQR) | - | 56 (42-65) | 126 (105-147) | <.0001 |
| Range | - | 28-98 | 57-187 |  |
| B. Diagnostic categories, n (%) |  |  |  |  |
| Diseases of the musculoskeletal system and connective tissue (DM00-DM94) | 187 (23) | 126 (24) | 51 (24) | 0.8837 |
| Injury, poisoning and certain other consequences of external causes (DS00-DT98) | 170 (21) | 119 (22) | 42 (20) | 0.5970 |
| Diseases of the nervous system (DG00-DG99) | 105 (13) | 58 (11) | 22 (10) | 0.4745 |
| Malignant neoplasms (DC00-DC97) and cancer-related medical care inducing neuropathic pain | 106 (13) | 46 (9) | 15 (7) | 0.0098 |
| Other diagnoses | 54 (7) | 34 (6) | 15 (7) | 0.9578 |
| Multiple diagnoses | 204 (25) | 146 (28) | 69 (32) | 0.0717 |
| C. Oral cannabinoid regimen prescribed at baseline |  |  |  |  |
| THC, n (%)  Dose (mg) median (IQR)  Dose (mg) range  Missing, n | **442 (54)**  -  -  0 | **284 (54)**  7.5 (7.47-14.9)  0.83-24.9  32 | **110 (51)**  10.4 (7.5-16.6)  1.66-34.86  14 | 0.8369  0.0081 |
| CBD, n (%)  Dose (mg) median (IQR)  Dose (mg) range  Missing, n | **319 (39)**  -  -  0 | **198 (37)**  33.4 (33.4-33.4)  3.34-125.25  17 | **82 (38)**  33.4 (25.05-33.4)  8.35-66.8  13 | 0.9067  0.3799 |
| THC/CBD, n (%)  THC dose (mg) median (IQR)  THC dose (mg) range  THC missing, n  CBD dose (mg) median (IQR)  CBD dose (mg) range  CBD missing, n | **65 (8)**  **-**  **-**  0  -  -  0 | **47 (9)**  7.06 (3.75-15.0)  0.83-40  5  31.73 (20.88-33.4)  1.67-50.1  7 | **22 (10)**  12.45 (6.23-17.02)  0.83-30  2  28.36 (20.86-33.4)  13.36-66.8  2 | 0.5001  0.1742  0.6009 |

SD (Standard deviation), BMI (Body mass index), IQR (Interquartile range), THC (Tetrahydrocannabinol); CBD (Cannabidiol).

Statistics: Chi^2^ (Gender; Diagnostic categories, type of CBM regimen), One-way ANOVA (Age; BMI), Kruskal Wallis (Days from baseline to follow-up; dose of oral cannabinoid regimens).

Table S3. Overview of adverse events reported in accordance with the different oral cannabinoid regimens at first follow-up consultation (N=529)

|  | THC  N=284  n (%) | CBD  N=198  n (%) | THC/CBD  N=47  n (%) | P-value | Total  N=529  n (%) |
| --- | --- | --- | --- | --- | --- |
| One or more adverse events | **145 (52)** | **59 (30)** | **19 (34)** | **<.0001** | **223 (42)** |
|  |  |  |  |  |  |
| Gastrointestinal disorders | **64 (23)** | **19 (10)** | **8 (17)** | **0.0011** | **91 (17)** |
| Dry mouth | 33 (12) | 13 (7) | 2 (4) | NA | 48 (9) |
| Increased appetite | 15 (5) | 1 (1) | 3 (6) | NA | 19 (4) |
| Nausea | 9 (3) | 0 | 2 (4) | NA | 11 (2) |
| Diarrhoea | 4 (1) | 2 (1) | 0 | NA | 6 (1) |
| Constipation | 3 (1) | 2 (1) | 0 | NA | 5 (1) |
| Abdominal pain | 4 (1) | 0 | 0 | NA | 4 (1) |
| Vomiting | 0 | 0 | 0 | NA | 0 |
| Decreased appetite | 1 (<1) | 0 | 0 | NA | 1 (<1) |
| Other gastrointestinal reactions^a^ | 1 (<1) | 2 (1) | 2 (4) | NA | 5 (1) |
|  |  |  |  |  |  |
| Nervous system disorders | **58 (20)** | **13 (7)** | **5 (11)** | **<.0001** | **76 (14)** |
| Dizziness | 36 (13) | 3 (2) | 3 (6) | NA | 42 (8) |
| Headache | 14 (5) | 5 (3) | 1 (2) | NA | 20 (4) |
| Cognitive disturbances | 5 (2) | 0 | 0 | NA | 5 (1) |
| Somnolence | 3 (1) | 1 (1) | 1 (2) | NA | 5 (1) |
| Sedation | 4 (1) | 1 (1) | 0 | NA | 5 (1) |
| Disorientation | 1 (<1) | 3 (2) | 0 | NA | 4 (1) |
| Other nervous system disorders^b^ | 1 (<1) | 0 | 0 | NA | 1 (<1) |
|  |  |  |  |  |  |
| General disorders and administration site conditions | **49 (17)** | **17 (8)** | **7 (15)** | **0.0245** | **71 (13)** |
| Fatigue | 48 (17) | 16 (8) | 7 (15) | NA | 71 (13) |
| Gait disturbance | 0 | 1 (1) | 0 | NA | 1 (<1) |
| Flu like symptoms | 2 (1) | 0 | 0 | NA | 2 (<1) |
|  |  |  |  |  |  |
| Psychiatric disorder | **17 (6)** | **7 (4)** | **0** | **0.1307** | **24 (5)** |
| Euphoria | 7 (2) | 2 (1) | 0 | NA | 9 (2) |
| Insomnia | 0 | 5 (3) | 0 | NA | 5 (1) |
| Depressive mood | 4 (1) | 0 | 0 | NA | 4 (1) |
| Agitation | 1 (<1) | 0 | 0 | NA | 1 (<1) |
| Mood swings | 1 (<1) | 0 | 0 | NA | 1 (<1) |
| Anxiety | 1 (<1) | 0 | 0 | NA | 1 (<1) |
| Confusion | 1 (<1) | 0 | 0 | NA | 1 (<1) |
| Psychosis | 1 (<1) | 0 | 0 | NA | 1 (<1)^c^ |
| Other psychiatric disorders^d^ | 1 (<1) | 0 | 0 | NA | 1 (<1) |
|  |  |  |  |  |  |
| Vascular disorders | **5 (2)** | **0** | **1 (2)** | NA | **6 (1)** |
| Increased perspiration/hot flushes | 5 (2) | 0 | 1 (2) | NA | 6 (1) |
|  |  |  |  |  |  |
| Musculoskeletal disorders | **1 (<1)** | **4 (2)** | **1 (2)** | NA | **6 (1)** |
| Muscle weakness | 1 (<1) | 3 (2) | 1 (2) | NA | 5 (1) |
| Musculoskeletal pain | 0 | 1 (1) | 0 | NA | 1 (<1) |
|  |  |  |  |  |  |
| Skin and subcutaneous tissue disorders | **1 (<1)** | **2 (1)** | **0** | NA | **3 (1)** |
| Skin irritation | 1 (<1) | 2 (1) | 0 | NA | 3 (1) |
|  |  |  |  |  |  |
| Eye disorders | 1 (<1) | 1 (1) | 0 | NA | 2 (<1) |
| Visual disturbance | 0 | 0 | 0 | NA | 0 |
| Eye irritation | 1 (<1) | 1 (1) | 0 | NA | 2 (<1) |
|  |  |  |  |  |  |
| Respiratory disorders | **0** | **1 (1)** | 0 | NA | **1 (<1)** |
| Cough | 0 | 1 (1) | 0 | NA | 1 (<1) |
| Other respiratory disorders | 0 | 0 | 0 | NA | 0 |
|  |  |  |  |  |  |
| Cardiac disorders | **0** | **1 (1)** | **0** | NA | **1 (<1)** |
| Palpitations of the heart | 0 | 1 (1) | 0 | NA | 1 (<1) |
|  |  |  |  |  |  |
| Sensory disorders | **0** | **0** | **1 (2)** | NA | **1 (<1)** |
| Sensory disturbances | 0 | 0 | 1 (2) | NA | 1 (<1) |
|  |  |  |  |  |  |
| Other disorders^e^ | **6 (2)** | **2 (1)** | **2 (4)** | NA | **10 (2)** |
| Missing, n | 1 | 0 | 0 |  | 1 |

^a^Other gastrointestinal disorders e.g., heartburn.

^b^Other nervous system disorders e.g., syncope.

^c^One patient (0.2%) developed hallucinations following intake of THC. The patient did not comply with the recommended dosage guideline.

^d^Other psychiatric disorders e.g., feeling claustrophobic.

^e^Other disorders e.g., felling unwell.

THC (Tetrahydrocannabinol); CBD (Cannabidiol).

Table S4. Overview of adverse events reported in accordance with the different oral cannabinoid regimens at second follow-up consultation (N=214)

|  | THC  N=110  n (%) | CBD  N=82  n (%) | THC/CBD  N=22  n (%) | P-value | Total  N=214  n (%) |
| --- | --- | --- | --- | --- | --- |
| One or more adverse events | **41 (37)** | **22 (27)** | **9 (41)** | **0.2021** | **72 (34)** |
|  |  |  |  |  |  |
| Gastrointestinal disorders | **16 (15)** | **9 (11)** | **2 (9)** | **0.5564** | **27 (13)** |
| Dry mouth | 8 (7) | 4 (5) | 1 (5) | NA | 13 (6) |
| Increased appetite | 5 (5) | 0 | 0 | NA | 5 (2) |
| Nausea | 1 (1) | 2 (2) | 0 | NA | 3 (1) |
| Diarrhoea | 1 (1) | 1 (1) | 0 | NA | 2 (1) |
| Constipation | 0 | 0 | 0 | NA | 0 |
| Abdominal pain | 0 | 0 | 0 | NA | 0 |
| Vomiting | 1 (1) | 0 | 0 | NA | 1 (<1) |
| Decreased appetite | 0 | 0 | 0 | NA | 0 |
| Other gastrointestinal reactions^a^ | 1 (1) | 2 (2) | 1 (5) | NA | 4 (2) |
|  |  |  |  |  |  |
| Nervous system disorders | **16 (15)** | **4 (5)** | **3 (14)** | **0.0912** | **23 (11)** |
| Dizziness | 9 (8) | 2 (2) | 1 (5) | NA | 12 (6) |
| Headache | 3 (3) | 2 (2) | 0 | NA | 5 (2) |
| Cognitive disturbances | 1 (1) | 0 | 1 (5) | NA | 2 (1) |
| Somnolence | 3 (1) | 0 | 0 | NA | 3 (1) |
| Sedation | 0 | 0 | 1 (5) | NA | 1 (<1) |
| Disorientation | 0 | 0 | 0 | NA | 0 |
| Other nervous system disorders^b^ | 0 | 0 | 1 (5) | NA | 1 (<1) |
|  |  |  |  |  |  |
| General disorders and administration site conditions | **14 (13)** | **4 (5)** | **2 (9)** | **0.1810** | **20 (9)** |
| Fatigue | 14 (13) | 4 (5) | 1 (5) | NA | 19 (9) |
| Gait disturbance | 0 | 0 | 0 | NA | 0 |
| Flu like symptoms | 0 | 0 | 1 (5) | NA | 1 (<1) |
|  |  |  |  |  |  |
| Psychiatric disorder | **4 (4)** | **2 (2)** | **0** | **0.8842** | **6 (3)** |
| Euphoria | 1 (1) | 0 | 0 | NA | 1 (<1) |
| Insomnia | 0 | 1 (1) | 0 | NA | 1 (<1) |
| Depressive mood | 0 | 0 | 0 | NA | 0 |
| Agitation | 1 (1) | 1 (1) | 0 | NA | 2 (1) |
| Mood swings | 0 | 0 | 0 | NA | 0 |
| Anxiety | 0 | 0 | 0 | NA | 0 |
| Confusion | 0 | 0 | 0 | NA | 0 |
| Psychosis | 0 | 0 | 0 | NA | 0 |
| Other psychiatric disorders^c^ | 2 (2) | 0 | 0 | NA | 2 (1) |
|  |  |  |  |  |  |
| Vascular disorders | **2 (2)** | **0** | **0** | NA | **2 (1)** |
| Increased perspiration/hot flushes | 2 (2) | 0 | 0 | NA | 2 (1) |
|  |  |  |  |  |  |
| Musculoskeletal disorders | **1 (1)** | **4 (5)** | **0** | NA | **5 (2)** |
| Muscle weakness | 1 (1) | 3 (4) | 0 | NA | 4 (2) |
| Musculoskeletal pain | 0 | 1 (1) | 0 | NA | 1 (<1) |
|  |  |  |  |  |  |
| Skin and subcutaneous tissue disorders | **0** | **1 (1)** | **0** | NA | **1 (<1)** |
| Skin irritation | 0 | 1 (1) | 0 | NA | 1 (<1) |
|  |  |  |  |  |  |
| Eye disorders | 0 | 0 | 1 (5) | NA | 1 (<1) |
| Visual disturbance | 0 | 0 | 1 (5) | NA | 1 (<1) |
| Eye irritation | 0 | 0 | 0 | NA | 0 |
|  |  |  |  |  |  |
| Respiratory disorders | **1 (1)** | **1 (1)** | 1 (5) | NA | **3 (1)** |
| Cough | 0 | 1 (1) | 1 (5) | NA | 2 (1) |
| Other respiratory disorders | 1 (1) | 0 | 0 | NA | 1 (<1) |
|  |  |  |  |  |  |
| Cardiac disorders | **1 (1)** | **0** | **0** | NA | **1 (<1)** |
| Palpitations of the heart | 1 (1) | 0 | 0 | NA | 1 (<1) |
|  |  |  |  |  |  |
| Sensory disorders | **0** | **0** | **0** | NA | **0** |
| Sensory disturbances | 0 | 0 | 0 | NA | 0 |
|  |  |  |  |  |  |
| Other disorders^d^ | **1 (1)** | **0** | **1 (4)** | NA | **2 (1)** |
| Missing, n | 0 | 1 | 0 | NA | 1 |

^a^Other gastrointestinal disorders e.g., heartburn.

^b^Other nervous system disorders e.g., syncope.

^c^Other psychiatric disorders e.g., feeling claustrophobic.

^d^Other disorders e.g., felling unwell.

THC (Tetrahydrocannabinol); CBD (Cannabidiol).

Table S5. First follow-up consultation demographic and clinical characteristics of patients with treatment refractory chronic pain (non-cancer-related versus cancer-related) receiving oral cannabinoid therapy (N=383)

|  | Pain | |  |
| --- | --- | --- | --- |
| A. Characteristics | **Non-cancer-related**  **n=337** | **Cancer-related**  **n=46** | **P-value** |
| Gender, n (%) |  |  |  |
| Female | 235 (70) | 32 (70) | 0.9815 |
| Male | 102 (30) | 14 (30) |  |
| Age |  |  |  |
| Mean years ±SD | 59 ±16.6 | 60 ±10.7 | 0.5305 |
| BMI |  |  |  |
| Mean ±SD | 25.9 ±5.8 | 24 ±4.4 | 0.0373 |
| Days from baseline to follow-up |  |  |  |
| Median (IQR) | 56 (42-65) | 47 (40-63) | 0.3106 |
| Range | 28-98 | 28-96 |  |
| B. Oral cannabinoid regimen prescribed at baseline |  |  |  |
| THC, n (%)  Dose (mg) median (IQR)  Dose (mg) range  Missing, n | **183 (54)**  7.5 (7.47-15.0)  0.83-24.9  22 | **32 (70)**  7.47 (7.06-14.94)  1.66-22.41  4 | 0.0504  0.0883 |
| CBD, n (%)  Dose (mg) median (IQR)  Dose (mg) range  Missing, n | **129 (38)**  33.4 (33.4-33.4)  3.34-50.1  15 | **5 (11)**  29.23 (20.88-33.4)  16.7-33.4  1 | 0.0003  0.1429 |
| THC/CBD, n (%)  THC dose (mg) median (IQR)  THC dose (mg) range  THC missing, n  CBD dose (mg) median (IQR)  CBD dose range  CBD missing, n | **25 (7)**  6.24 (3.32-15)  0.83-40  3  31.7 (20.04-33.4)  7.5-50.1  5 | **9 (20)**  12.45 (7.47-22.41)  2.49-22.41  2  33.4 (21.71-33.4)  16.7-33.4  2 | 0.0066  0.3192  0.5623 |
| C. Adverse events |  |  |  |
| One or more adverse reactions, n (%) | **139 (41)** | **22 (48)** | 0.3964 |
| Gastrointestinal disorders | 56 (17) | 12 (26) | 0.1149 |
| Nervous system disorders | 51 (15) | 2 (4) | 0.0469 |
| General disorders and administration site conditions | 44 (13) | 8 (17) | 0.4208 |
| Psychiatric disorder | 16 (5) | 4 (9) | 0.2589 |
| Vascular disorders | 6 (2) | 0 | NA |
| Musculoskeletal disorders | 5 (1) | 0 | NA |
| Skin and subcutaneous tissue disorders | 1 (<1) | 0 | NA |
| Eye disorders | 1 (<1) | 0 | NA |
| Sensory disorders | 0 | 0 | NA |
| Cardiac disorders | 1 (<1) | 0 | NA |
| Respiratory disorders | 0 | 0 | NA |
| Other disorders | 6 (2) | 0 | NA |
| Missing, n | 1 | 0 |  |
| D. NRS, collectively mean of mean ±SD |  |  |  |
| Baseline consultation | 7.1 ±1.6 | 5.8 ±2.0 | <.0001 |
| First follow-up consultation | 5.9±2.2 | 3.1±2.5 | <.0001 |
| P-value | <.0001 | <.0001 | <.0001 |
| Mean reduction NRS from baseline to follow-up | 1.3±2.1 | 2.5±2.7 | 0.0026 |
| Missing, n | 67 | 10 |  |
| E. Percentage change in paired mean NRS n (%) |  |  |  |
| Increase NRS | 49 (18) | 5 (14) | 0.5289 |
| No change NRS | 47 (17) | 2 (6) | 0.0685 |
| Reduction NRS>0 - <30% | 98 (36) | 6 (17) | 0.0195 |
| Reduction NRS ≥30% - <50% | 32 (12) | 8 (22) | 0.0829 |
| Reduction NRS ≥50% | 44 (16) | 15 (42) | 0.0003 |
| Missing, n | 67 | 10 |  |

Non-cancer-related pain covers Diseases of the musculoskeletal system and connective tissue (DM00-DM94) n=126, Injury, poisoning and certain other consequences of external causes (DS00-DT98) n=119, Diseases of the nervous system (DG00-DG99) n=58 and Other diagnoses n=34.

SD (Standard deviation), BMI (Body mass index), IQR (Interquartile range), THC (Tetrahydrocannabinol); CBD (Cannabidiol); NRS (Numeric rating scale).

Statistics: Chi^2^ (Gender; Diagnostic categories, type of CBM regimen, Adverse events, Percentage change in NRS), Two sample t-test (Age; BMI; NRS difference between CBM regimens), Kruskal Wallis (Days from baseline to follow-up; dose of oral cannabinoid regimens), Paired t-test (NRS difference between baseline and follow-up).
